# Supplementary material for: Metabolite Profiling and Distribution of Militarine in Rats Using UPLC-Q-TOF-MS/MS
Source: Molecules. 2020 Feb 28;25(5):1082. doi: 10.3390/molecules25051082 (PMC7179186; doi:10.3390/molecules25051082)
Supplement: Supplementary file 1 [file molecules-25-01082-s001.pdf]

Supplementary materials

Metabolites profiling and distribution of Militarine in rats by ultra-performance liquid chromatography coupled with quadrupole-TOF-MS/MS

Limin Li [1], Bin Hao[2], Yulong Zhang[2], Shen Ji[3], Guixin Chou[1*]

[1] Institute of Chinese Materia Medica, Shanghai University of Traditional Chinese Medicine, Shanghai 201203, China; lilimin_sifdc@163.com (L. L.);
[2] School of Pharmacy, Shanghai JiaoTong University, Shanghai 200240, China; bhao@sjtu.edu.cn (B.H.), yulong_zhang@sjtu.edu.cn（Y. Z）
[3] Shanghai Institute for Food and Drug Control, Shanghai 201203, China; jishen2013@163.com (S.J)

* Correspondence: chouguixinzyb@126.com (G.C); Tel.: +86-021-50271706 (G.C)

Contents of Supplementary Information

No.	Contents	Pages
1.	**Table S1** The custom fragment ions and neutral-loss molecules were used to screen the structurally related metabolites	5
2.	**Figure S1.** MS and MS/MS spectra of metabolite (M1) in negative ESI mode and the proposed fragmentation pathway.	6
3.	**Figure S2.** MS and MS/MS spectra of metabolite (M2) in negative ESI mode and the proposed fragmentation pathway.	6
4.	**Figure S3.** MS and MS/MS spectra of metabolite (M3) in negative ESI mode and the proposed fragmentation pathway.	6
5.	**Figure S4.** MS and MS/MS spectra of metabolite (M4) in negative ESI mode and the proposed fragmentation pathway.	7
6.	**Figure S5.** MS and MS/MS spectra of metabolite (M5) in negative ESI mode and the proposed fragmentation pathway.	7
7.	**Figure S6.** MS and MS/MS spectra of metabolite (M6) in negative ESI mode and the proposed fragmentation pathway.	7
8.	**Figure S7.** MS and MS/MS spectra of metabolite (M7) in negative ESI mode and the proposed fragmentation pathway.	8
9.	**Figure S8.** MS and MS/MS spectra of metabolite (M8) in negative ESI mode and the proposed fragmentation pathway.	8
10.	**Figure S9.** MS and MS/MS spectra of metabolite (M9) in negative ESI mode and the proposed fragmentation pathway.	8
11.	**Figure S10.** MS and MS/MS spectra of metabolite (M10) in negative ESI mode and the proposed fragmentation pathway.	9
12.	**Figure S11.** MS and MS/MS spectra of metabolite (M11) in negative ESI mode and the proposed fragmentation pathway.	9
13.	**Figure S12.** MS and MS/MS spectra of metabolite (M12) in negative ESI mode and the proposed fragmentation pathway.	9
14.	**Figure S13.** MS and MS/MS spectra of metabolite (M13) in negative ESI mode and the proposed fragmentation pathway.	10
15.	**Figure S14.** MS and MS/MS spectra of metabolite (M14) in negative ESI mode and the proposed fragmentation pathway.	10
16.	**Figure S15.** MS and MS/MS spectra of metabolite (M15) in negative ESI mode and the proposed fragmentation pathway.	10
17.	**Figure S16.** MS and MS/MS spectra of metabolite (M16) in negative ESI mode and the proposed fragmentation pathway.	11
18.	**Figure S17.** MS and MS/MS spectra of metabolite (M17) in negative ESI mode and the proposed fragmentation pathway.	11
19.	**Figure S18.** MS and MS/MS spectra of metabolite (M18) in negative ESI mode and the proposed fragmentation pathway.	11
20.	**Figure S19.** MS and MS/MS spectra of metabolite (M19) in negative ESI mode and the proposed fragmentation pathway.	12
21.	**Figure S20.** MS and MS/MS spectra of metabolite (M20) in negative ESI mode	12

	and the proposed fragmentation pathway.	
22.	**Figure S21.** MS and MS/MS spectra of metabolite (M21) in negative ESI mode and the proposed fragmentation pathway.	12
23.	**Figure S22.** MS and MS/MS spectra of metabolite (M22) in negative ESI mode and the proposed fragmentation pathway.	13
24.	**Figure S23.** MS and MS/MS spectra of metabolite (M23) in negative ESI mode and the proposed fragmentation pathway.	13
25.	**Figure S24.** MS and MS/MS spectra of metabolite (M24) in negative ESI mode and the proposed fragmentation pathway.	13
26.	**Figure S25.** MS and MS/MS spectra of metabolite (M25) in negative ESI mode and the proposed fragmentation pathway.	14
27.	**Figure S26.** MS and MS/MS spectra of metabolite (M26) in negative ESI mode and the proposed fragmentation pathway.	14
28.	**Figure S27.** MS and MS/MS spectra of metabolite (M27) in negative ESI mode and the proposed fragmentation pathway.	14
29.	**Figure S28.** MS and MS/MS spectra of metabolite (M28) in negative ESI mode and the proposed fragmentation pathway.	15
30.	**Figure S29.** MS and MS/MS spectra of metabolite (M29–M31) in negative ESI mode and the proposed fragmentation pathway.	15
31.	**Figure S30.** MS and MS/MS spectra of metabolite (M32) in negative ESI mode and the proposed fragmentation pathway.	16
32.	**Figure S31.** MS and MS/MS spectra of metabolite (M33, M37) in negative ESI mode and the proposed fragmentation pathway.	16
33.	**Figure S32.** MS and MS/MS spectra of metabolite (M34, M36) in negative ESI mode and the proposed fragmentation pathway.	17
34.	**Figure S33.** MS and MS/MS spectra of metabolite (M35, M38, M41, M44, M46, M49) in negative ESI mode and the proposed fragmentation pathway.	18
35.	**Figure S34.** MS and MS/MS spectra of metabolite (M39, M42, M43, M45, M47, M50, M54) in negative ESI mode and the proposed fragmentation pathway.	19
36.	**Figure S35.** MS and MS/MS spectra of metabolite (M40, M48, M53) in negative ESI mode and the proposed fragmentation pathway.	20
37.	**Figure S36.** MS and MS/MS spectra of metabolite (M51, M56) in negative ESI mode and the proposed fragmentation pathway.	21
38.	**Figure S37.** MS and MS/MS spectra of metabolite (M52, M55) in negative ESI mode and the proposed fragmentation pathway.	21
38.	**Figure S38.** MS and MS/MS spectra of metabolite (M57–M60) in negative ESI mode and the proposed fragmentation pathway.	22
40.	**Figure S39.** MS and MS/MS spectra of metabolite (M61, M66) in negative ESI mode and the proposed fragmentation pathway.	23
41.	**Figure S40.** MS and MS/MS spectra of metabolite (M62–M63) in negative ESI mode and the proposed fragmentation pathway.	24
42.	**Figure S41.** MS and MS/MS spectra of metabolite (M64–M65) in negative ESI mode and the proposed fragmentation pathway.	25
42.	**Figure S42.** MS and MS/MS spectra of metabolite (M67–M69) in negative ESI	26

	mode and the proposed fragmentation pathway.	
43.	**Figure S43** MS and MS/MS spectra of metabolite (M70–M71) in negative ESI mode and the proposed fragmentation pathway	27

1. The fragment ions were used to screen the structurally related metabolites with MS-Dial software.

Table S1. The custom fragment ions and neutral-loss molecules were used to screen the structurally related metabolites.

Fragment	m/z	Composition	Metabolites ID.
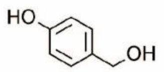	105.0346<br>123.0452	$C_7H_5O^-$<br>$C_7H_7O_2^-$	M0, M1, M4, M11, M12, M19, M22, M51, M56, M71
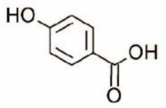	93.0346<br>137.0244	$C_6H_5O^-$<br>$C_7H_5O_3^-$	M2, M3, M6, M7, M8, M10, M13, M14, M15, M16, M20, M21, M24, M27, M28
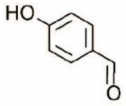	92.0268<br>121.0295	$C_6H_4O^-$<br>$C_7H_5O_2^-$	M6, M8, M17, M23, M26, M28
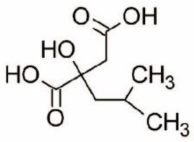	99.0815<br>127.0765<br>129.0557<br>189.0768	$C_6H_{11}O^-$<br>$C_7H_{11}O_2^-$<br>$C_6H_9O_3^-$<br>$C_8H_{13}O_5^-$	M0, M29-M33, M39, M40, M42, M43, M45, M47, M48, M50-M52, M54-M57, M59, M60, M62, M63, M70, M71
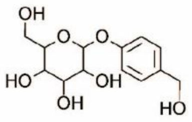	105.0346<br>123.0452<br>285.098	$C_7H_5O^-$<br>$C_7H_7O_2^-$<br>$C_{13}H_{17}O_7^-$	M0, M1, M4, M11, M12, M19, M22, M33, M34, M37, M40, M43, M48, M51-M53, M56, M61-M63, M70, M71
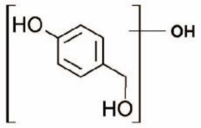	139.0401	$C_7H_7O_3^-$	M5, M6, M8, M10, M15, M62, M63
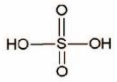	79.9574<br>96.9601	SO_3H^-<br>HSO_4^-	M1, M6, M8, M9, M11, M13, M18, M25, M57, M58, M59, M60
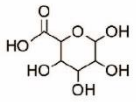	175.0248	$C_6H_7O_6^-$	M2, M3, M4, M5, M7, M16, M17, M22, M24, M33, M37, M52, M55, M61, M66
Di-Glc	323.0984	$C_{12}H_{19}O_{10}^-$	M35, M38, M39, M41, M42, M43, M44, M45, M46, M47, M49, M50, M54, M64, M65, M67, M68
Tri-Glc	485.1512	$C_{18}H_{29}O_{15}^-$	M35, M38, M41, M44, M46, M49
Glc+GA	337.0776	$C_{12}H_{17}O_{11}^-$	M22, M24, M40, M48, M53
Glc+SO3	241.0024	$C_6H_9O_8S^-$	M9, M13, M18, M25, M58, M57, M59, M60, M57, M58, M59, M60
Glc	162.0534	$C_6H_{11}O_5$	
Gastrodin	268.0952	$C_{13}H_{17}O_6$	
GluA	176.0326	$C_6H_8O_6$	

2. The MS and MS/MS of 71 metabolites in rats (Figure S1–S43)

(1) metabolite (M1) having the skeleton of p-hydroxybenzyl alcohol

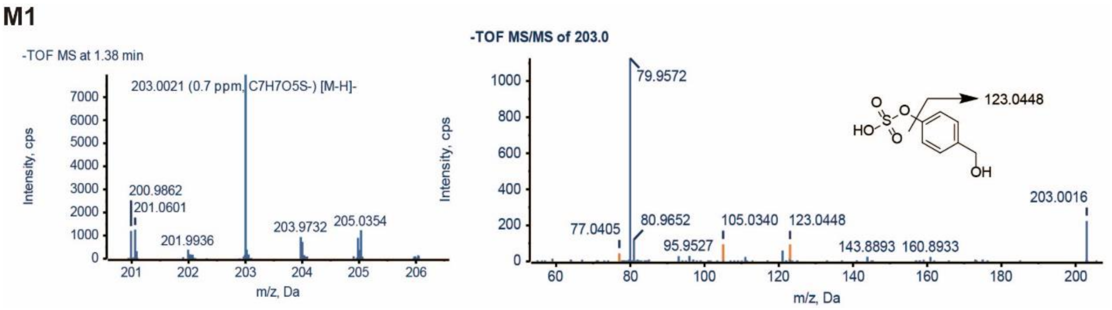

Figure S1. MS and MS/MS spectra of metabolite (M1) in negative ESI mode and the proposed fragmentation pathway.

(2) metabolite (M2) having the skeleton of p-hydroxybenzoic acid and GAS-COOH

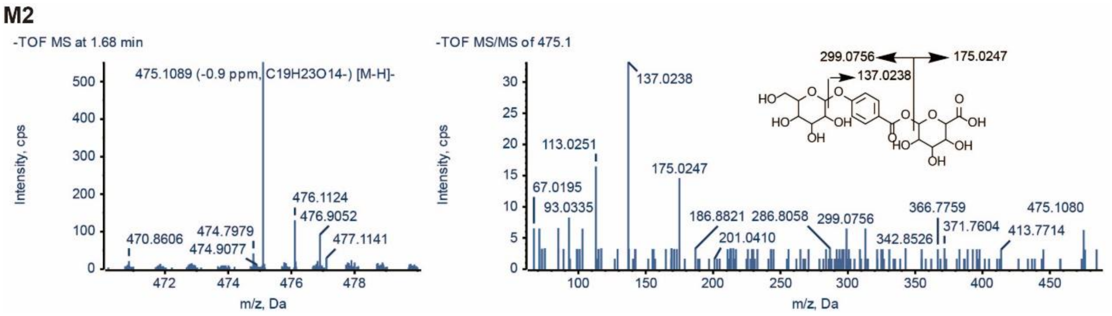

Figure S2. MS and MS/MS spectra of metabolite (M2) in negative ESI mode and the proposed fragmentation pathway.

(3) metabolite (M3) having the skeleton of p-hydroxybenzoic acid

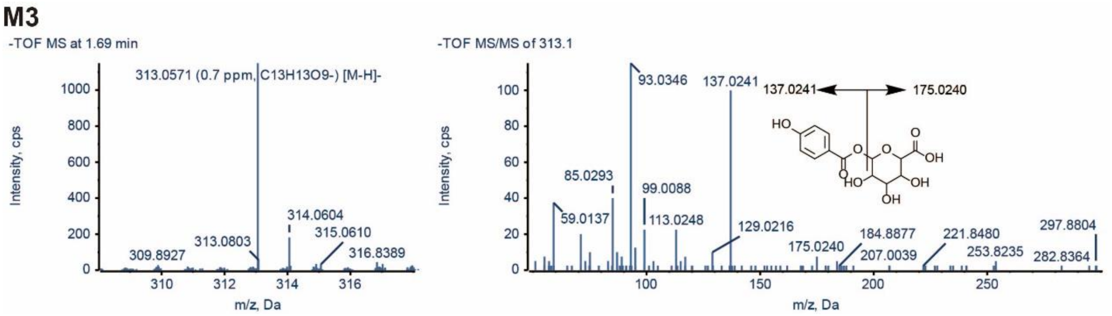

Figure S3. MS and MS/MS spectra of metabolite (M3) in negative ESI mode and the proposed fragmentation pathway.

(4) metabolite (M4) having the skeleton of p-hydroxybenzyl alcohol

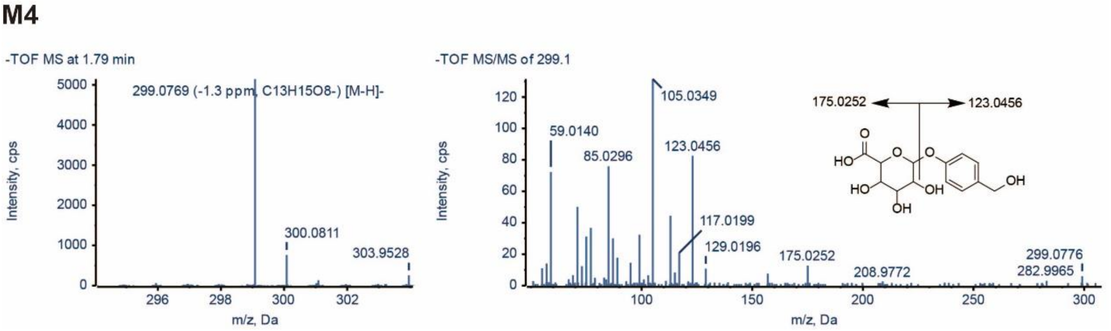

Figure S4. MS and MS/MS spectra of metabolite (M4) in negative ESI mode and the proposed fragmentation pathway.

(5) metabolite (M5) having the skeleton of oxidized p-hydroxybenzyl alcohol

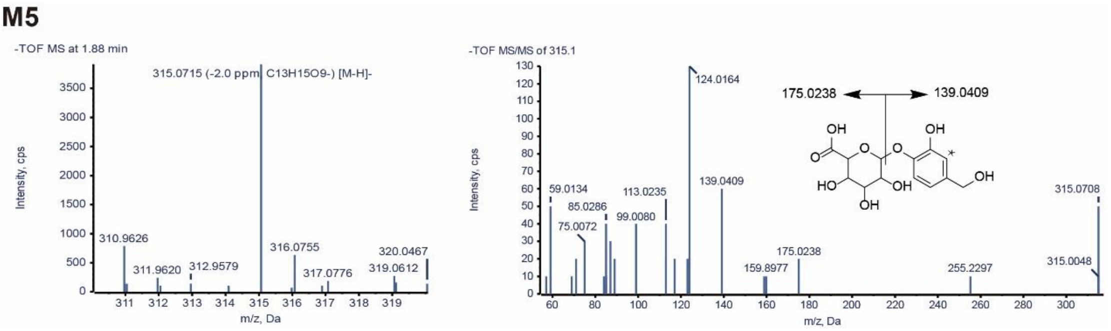

Figure S5. MS and MS/MS spectra of metabolite (M5) in negative ESI mode and the proposed fragmentation pathway. * presents the alternative position of hydroxyl.

(6) metabolite (M6) having the skeleton of oxidized p-hydroxybenzyl alcohol

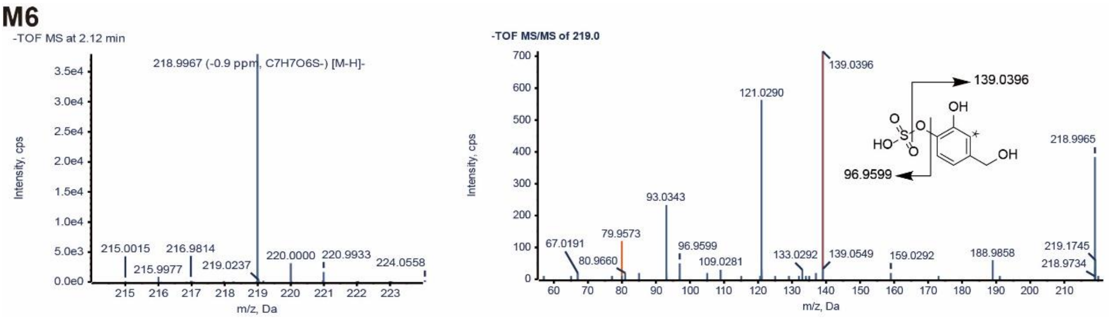

Figure S6. MS and MS/MS spectra of metabolite (M6) in negative ESI mode and the proposed fragmentation pathway. * presents the alternative position of hydroxyl.

(7) metabolite (M7) having the skeleton of p-hydroxybenzoic acid and GAS-COOH

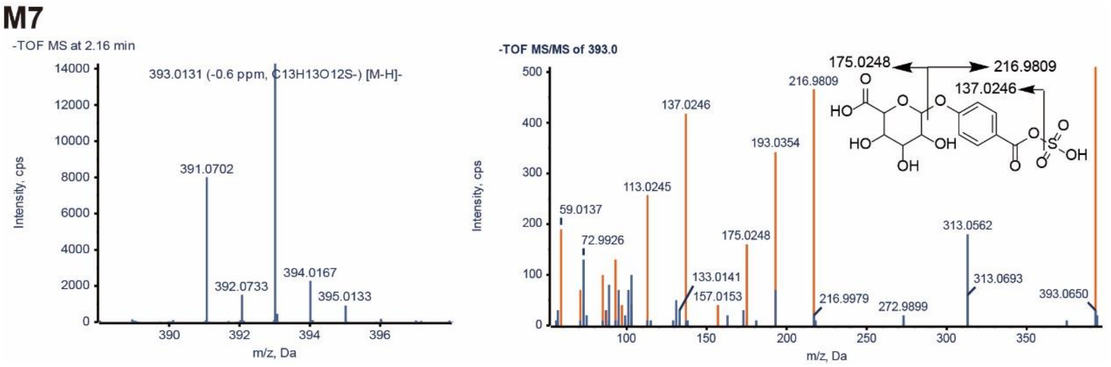

Figure S7. MS and MS/MS spectra of metabolite (M7) in negative ESI mode and the proposed fragmentation pathway.

(8) metabolite (M8) having the skeleton of hydroxylated p-hydroxybenzyl alcohol

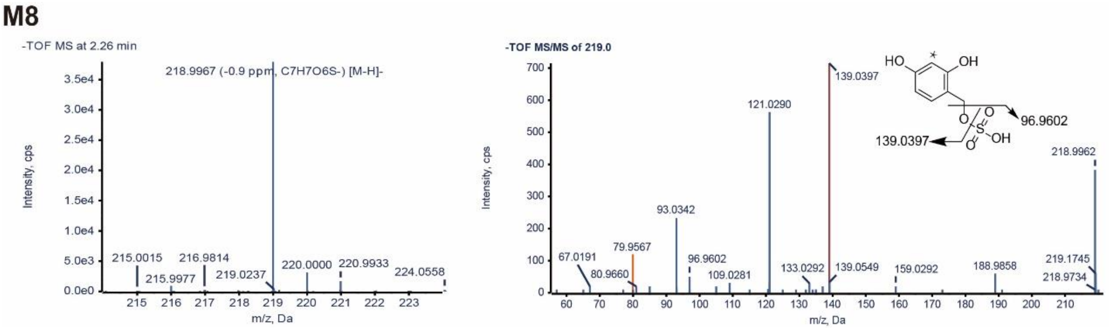

Figure S8. MS and MS/MS spectra of metabolite (M8) in negative ESI mode and the proposed fragmentation pathway. * presents the alternative position of hydroxyl.

(9) metabolite (M9) having the fragment of SO_3^-

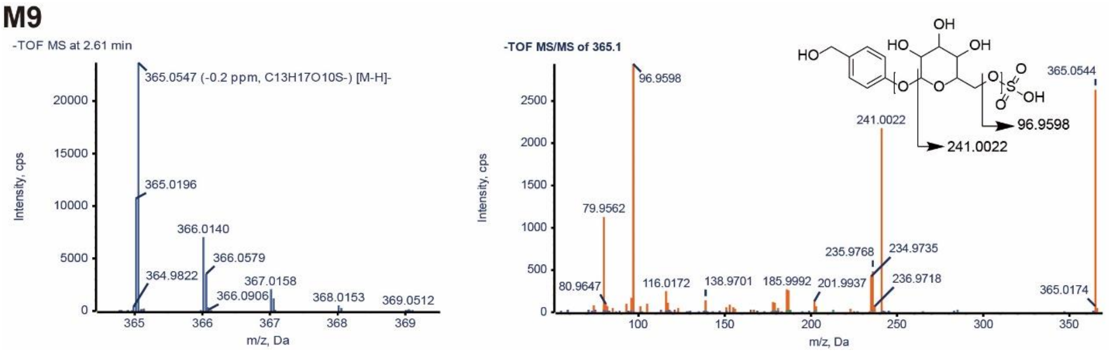

Figure S9. MS and MS/MS spectra of metabolite (M9) in negative ESI mode and the proposed fragmentation pathway.

(10) metabolite (M10) having the skeleton of hydroxylated p-hydroxybenzyl alcohol

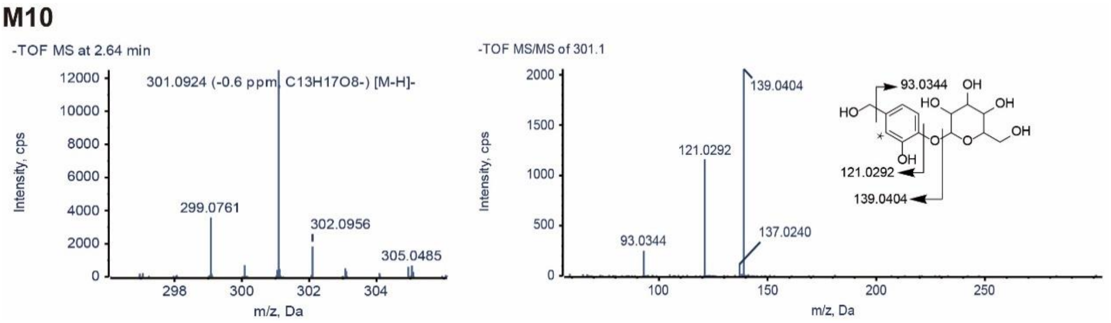

Figure S10. MS and MS/MS spectra of metabolite (M10) in negative ESI mode and the proposed fragmentation pathway. * presents the alternative position of hydroxyl.

(11) metabolite (M11) having the skeleton of p-hydroxybenzyl alcohol

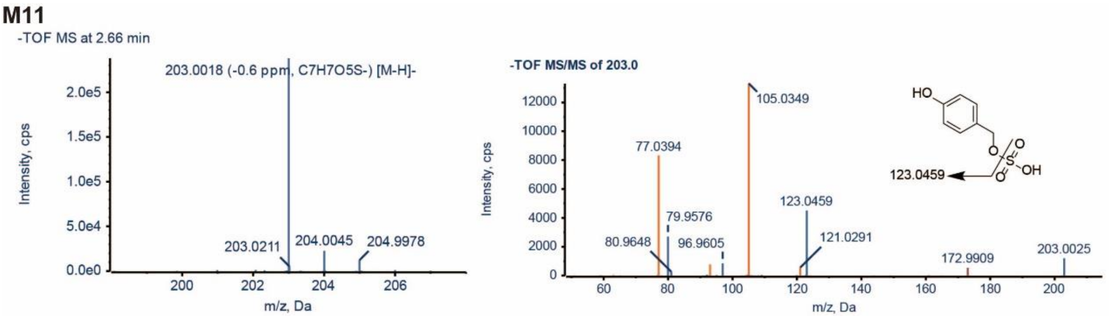

Figure S11. MS and MS/MS spectra of metabolite (M11) in negative ESI mode and the proposed fragmentation pathway.

(12) metabolite (M12) having the skeleton of p-hydroxybenzyl alcohol

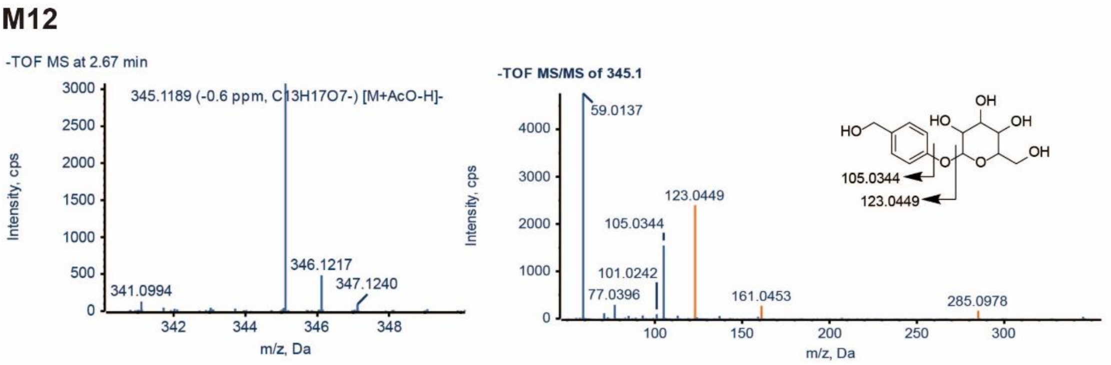

Figure S12. MS and MS/MS spectra of metabolite (M12) in negative ESI mode and the proposed fragmentation pathway.

(13) metabolite (M13) having the skeleton of p-hydroxybenzoic acid and gastrodin-COOH

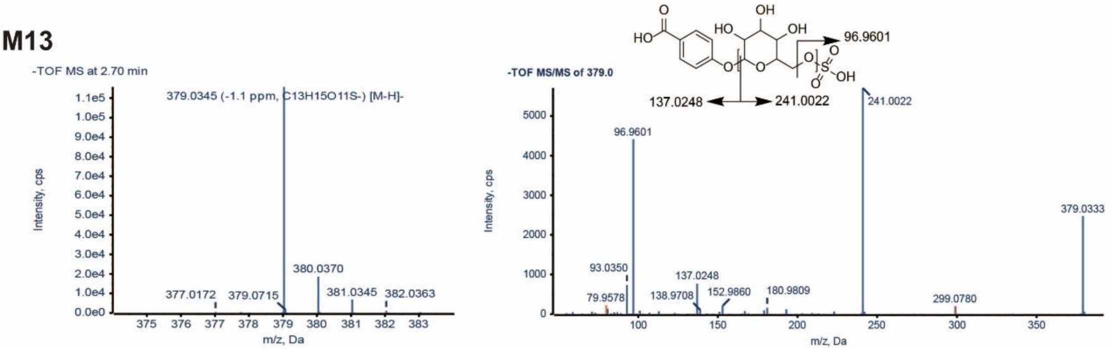

Figure S13. MS and MS/MS spectra of metabolite (M13) in negative ESI mode and the proposed fragmentation pathway.

(14) metabolite (M14) having the skeleton of p-hydroxybenzoic acid

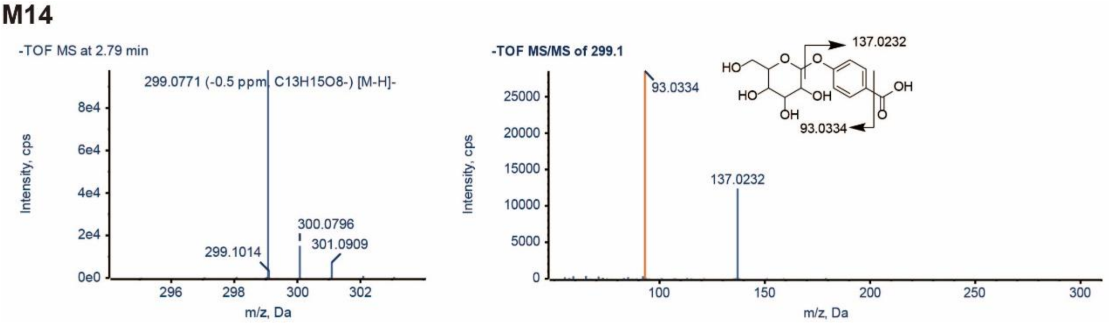

Figure S14. MS and MS/MS spectra of metabolite (M14) in negative ESI mode and the proposed fragmentation pathway.

(15) metabolite (M15) having the skeleton of hydroxylated p-hydroxybenzyl alcohol

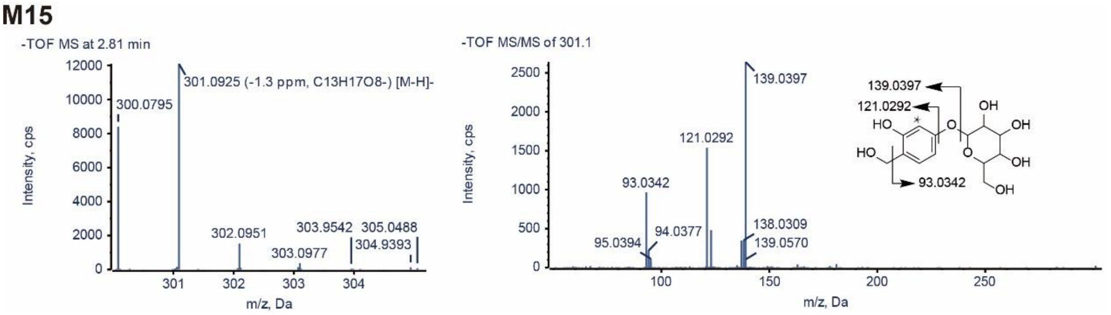

Figure S15. MS and MS/MS spectra of metabolite (M15) in negative ESI mode and the proposed fragmentation pathway. * presents the alternative position of hydroxyl.

(16) metabolite (M16) having the skeleton of p-hydroxybenzoic acid

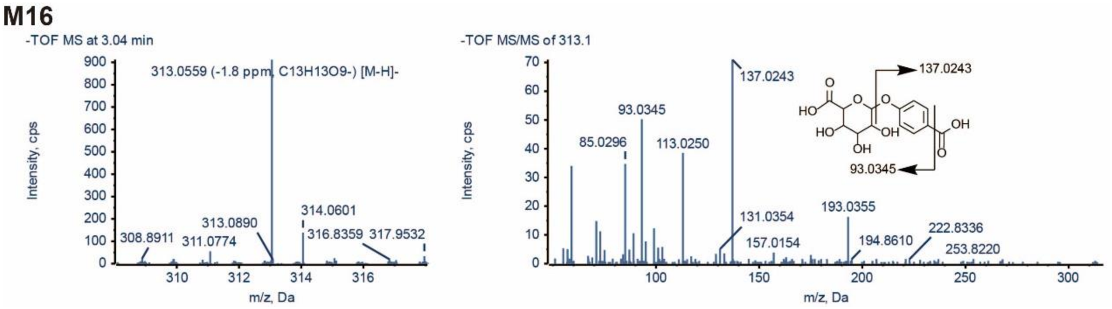

Figure S16. MS and MS/MS spectra of metabolite (M16) in negative ESI mode and the proposed fragmentation pathway.

(17) metabolite (M17) having the skeleton of p-hydroxybenzaldehyde

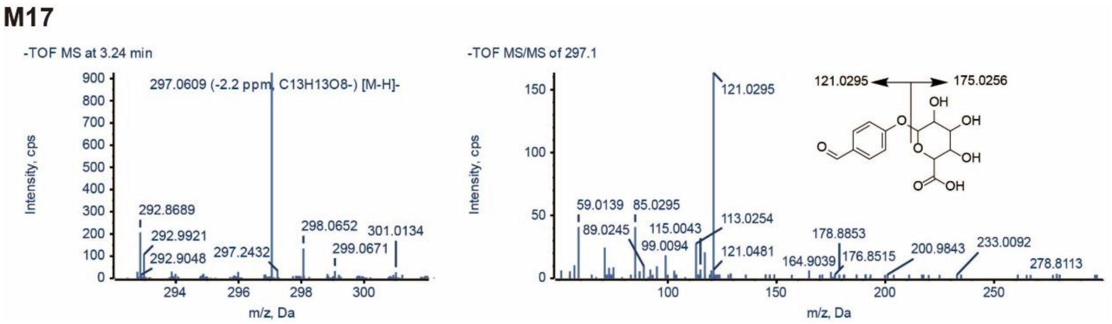

Figure S17. MS and MS/MS spectra of metabolite (M17) in negative ESI mode and the proposed fragmentation pathway.

(18) metabolite (M18) having the fragment of sulfated glucose

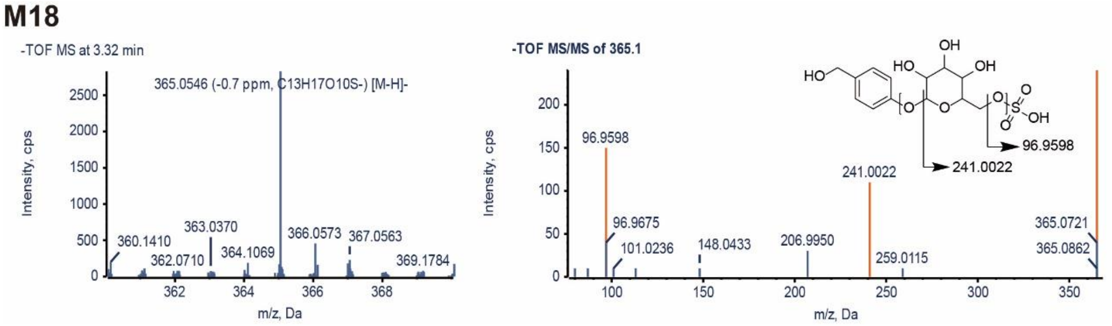

Figure S18. MS and MS/MS spectra of metabolite (M18) in negative ESI mode and the proposed fragmentation pathway.

(19) metabolite (M19) having the skeleton of p-hydroxybenzyl alcohol

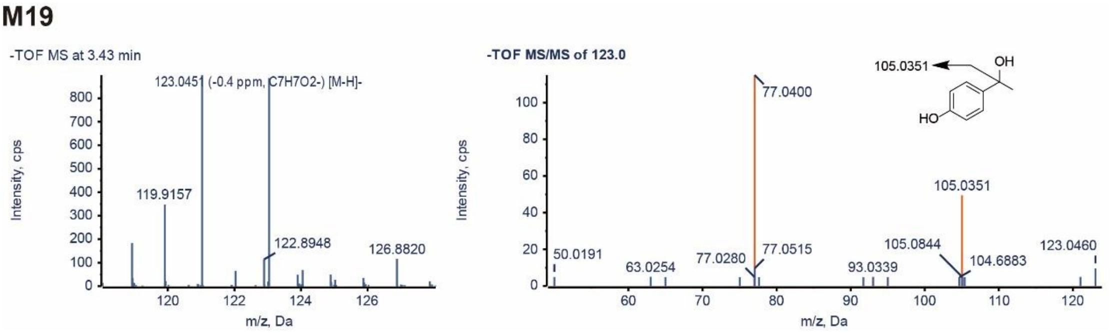

Figure S19. MS and MS/MS spectra of metabolite (M19) in negative ESI mode and the proposed fragmentation pathway.

(20) metabolite (M20) having the skeleton of p-hydroxybenzoic acid

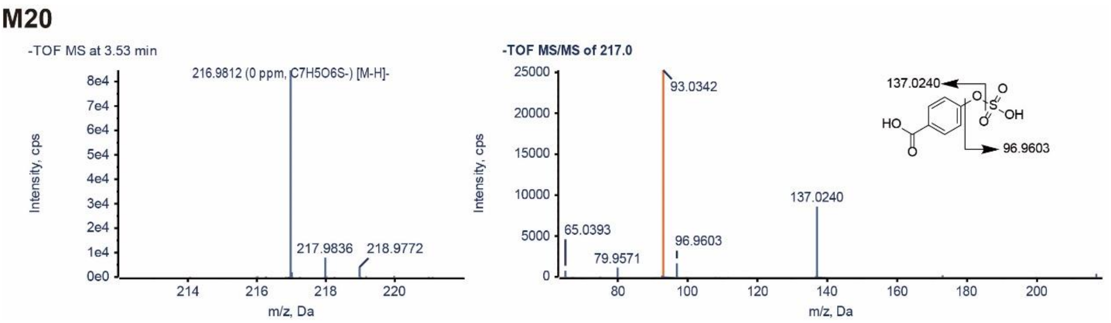

Figure S20. MS and MS/MS spectra of metabolite (M20) in negative ESI mode and the proposed fragmentation pathway.

(21) metabolite (M21) having the skeleton of p-hydroxybenzoic acid

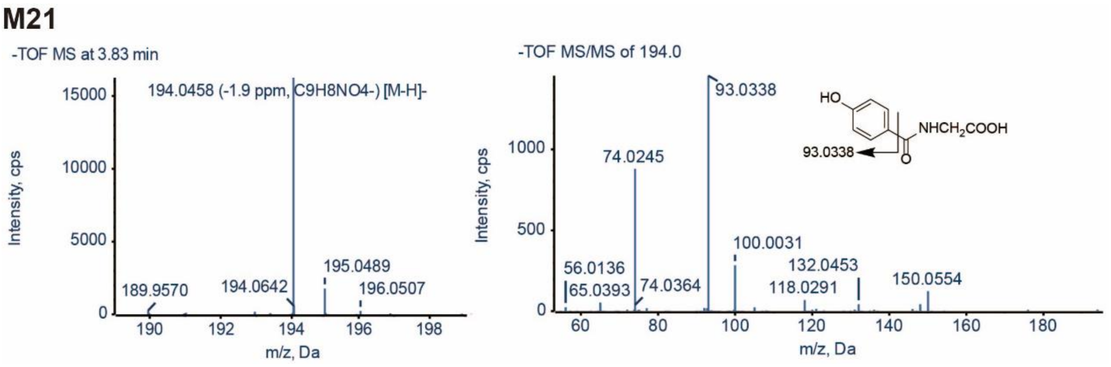

Figure S21. MS and MS/MS spectra of metabolite (M21) in negative ESI mode and the proposed fragmentation pathway.

(22) metabolite (M22) having the fragment of glucuronidated glucose

M22

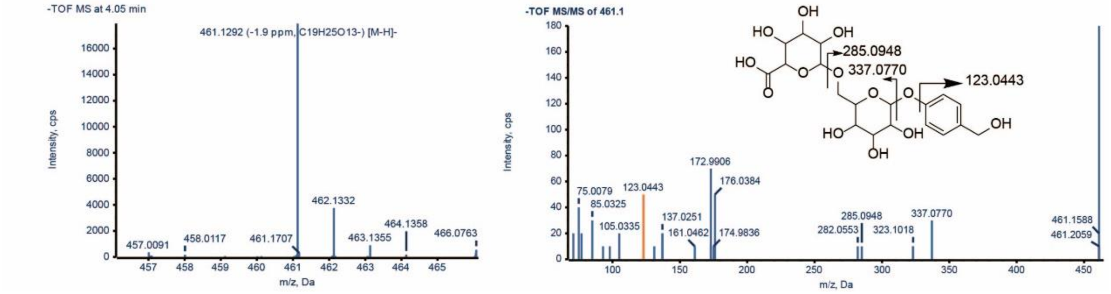

Figure S22. MS and MS/MS spectra of metabolite (M22) in negative ESI mode and the proposed fragmentation pathway.

(23) metabolite (M23) having the skeleton of p-hydroxybenzaldehyde

M23

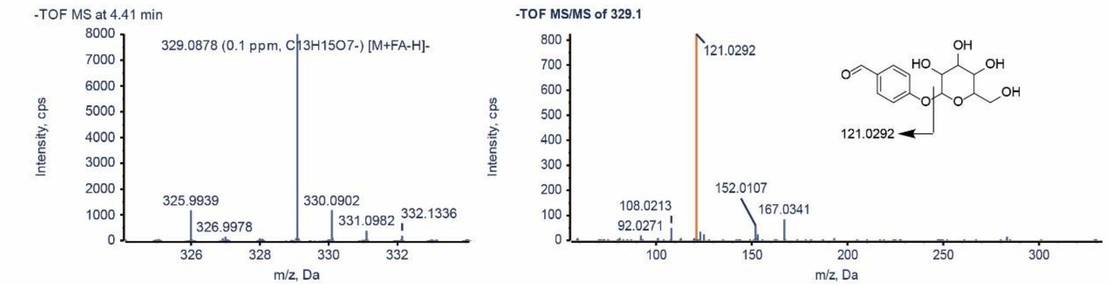

Figure S23. MS and MS/MS spectra of metabolite (M23) in negative ESI mode and the proposed fragmentation pathway.

(24) metabolite (M24) having the skeleton of p-hydroxybenzoic acid and the fragment of glucuronidated glucose

M24

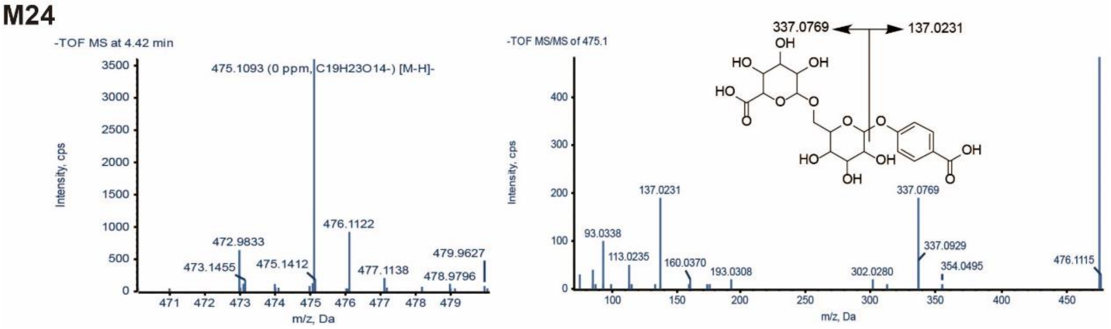

Figure S24. MS and MS/MS spectra of metabolite (M24) in negative ESI mode and the proposed fragmentation pathway.

(25) metabolite (M25) having the skeleton of p-hydroxybenzoic acid

M25

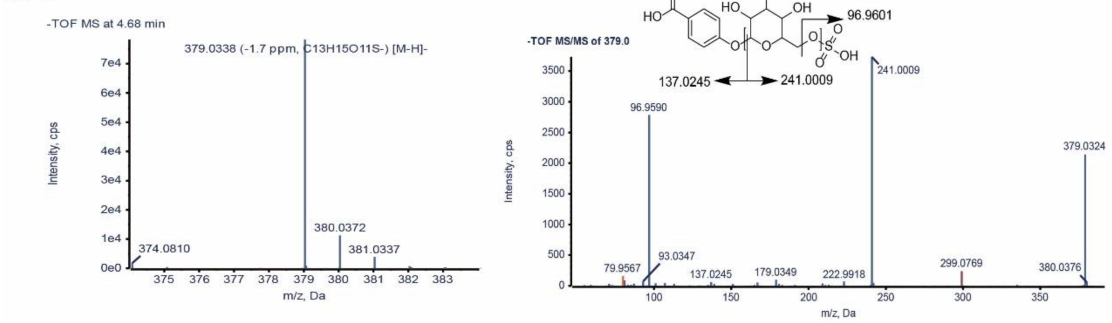

Figure S25. MS and MS/MS spectra of metabolite (M25) in negative ESI mode and the proposed fragmentation pathway.

(26) metabolite (M26) having the skeleton of p-hydroxybenzaldehyde

M26

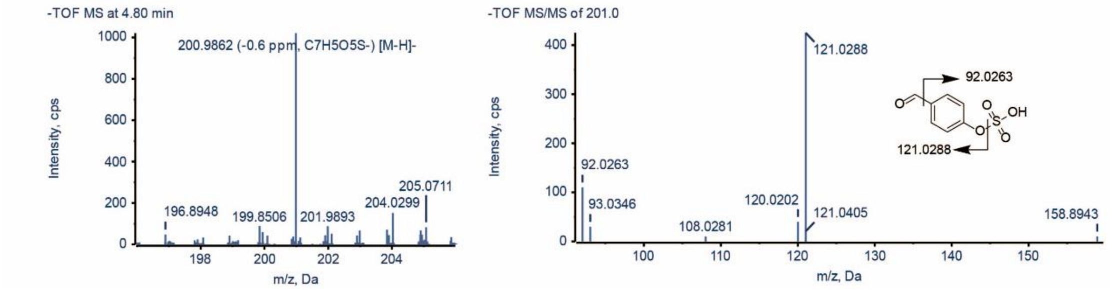

Figure S26. MS and MS/MS spectra of metabolite (M26) in negative ESI mode and the proposed fragmentation pathway.

(27) metabolite (M27) having the skeleton of p-hydroxybenzoic acid

M27

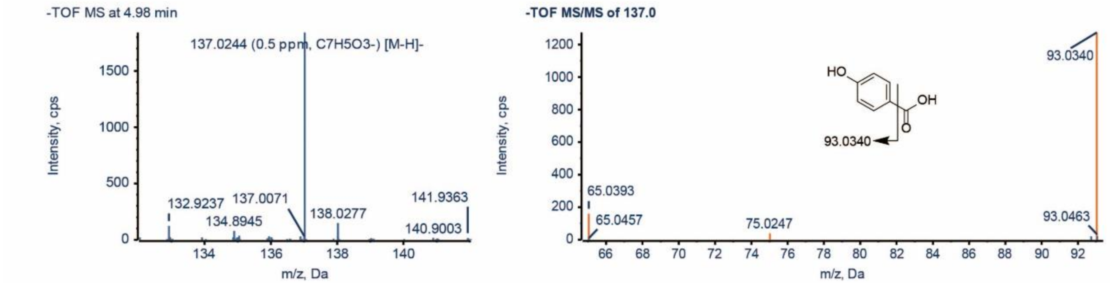

Figure S27. MS and MS/MS spectra of metabolite (M27) in negative ESI mode and the proposed fragmentation pathway.

(28) metabolite (M28) having the skeleton of p-hydroxybenzaldehyde

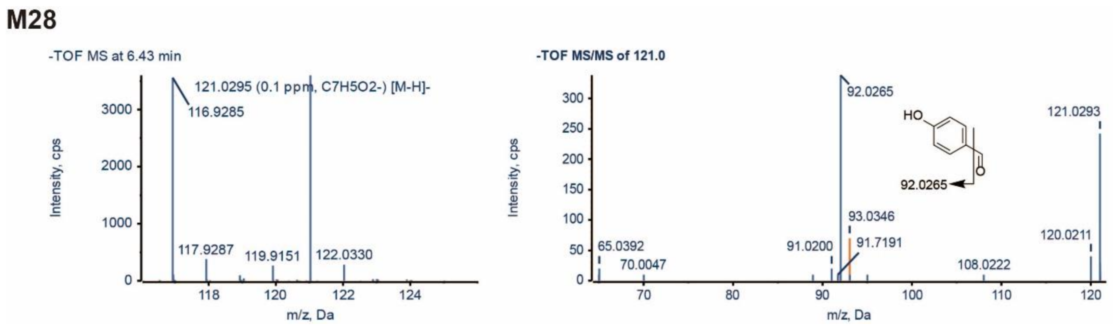

Figure S28. MS and MS/MS spectra of metabolite (M28) in negative ESI mode and the proposed fragmentation pathway.

(29) metabolites (M29 – M31) having the skeleton of isobutyl-malic acid

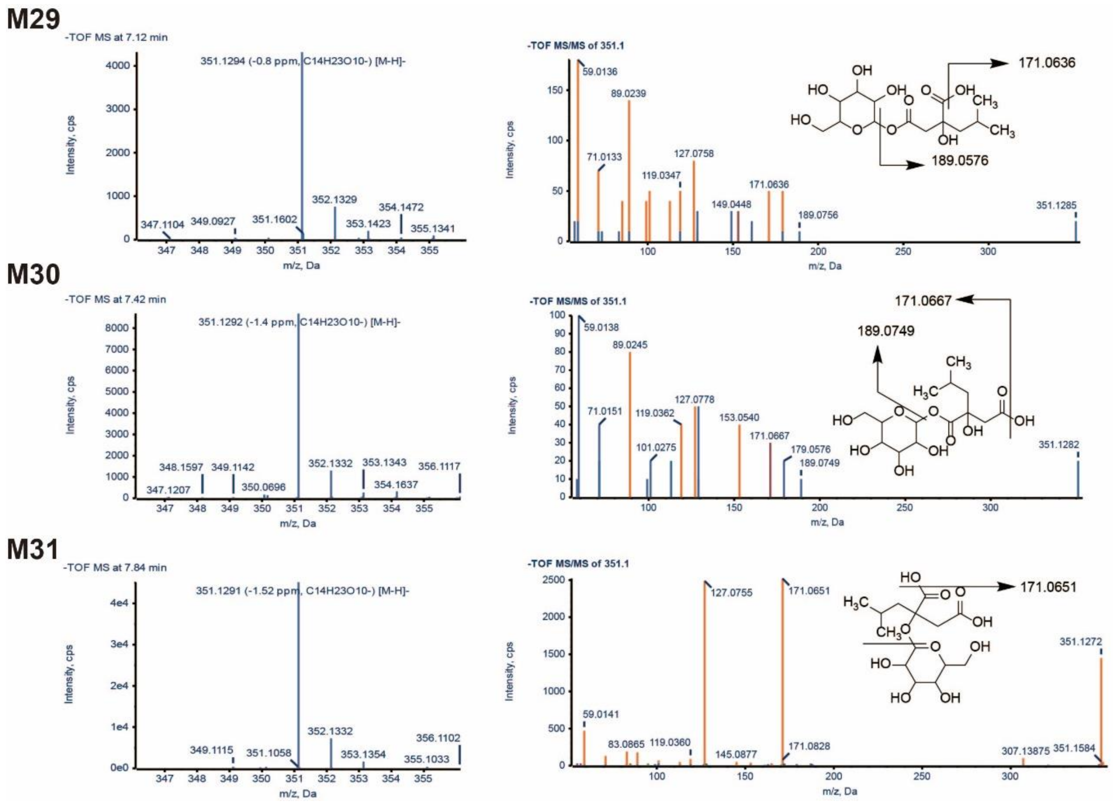

Figure S29. MS and MS/MS spectra of metabolites (M29 – M31) in negative ESI mode and the proposed fragmentation pathway.

(30) metabolite (M32) having the skeleton of isobutyl-malic acid

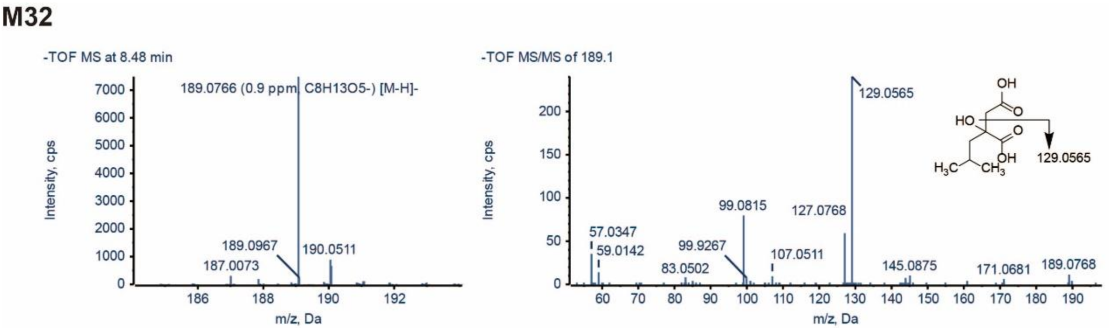

Figure S30. MS and MS/MS spectra of metabolite (M32) in negative ESI mode and the proposed fragmentation pathway.

(31) metabolites (M33, M37) having the skeleton of gastrodin and gymnoside I(II)

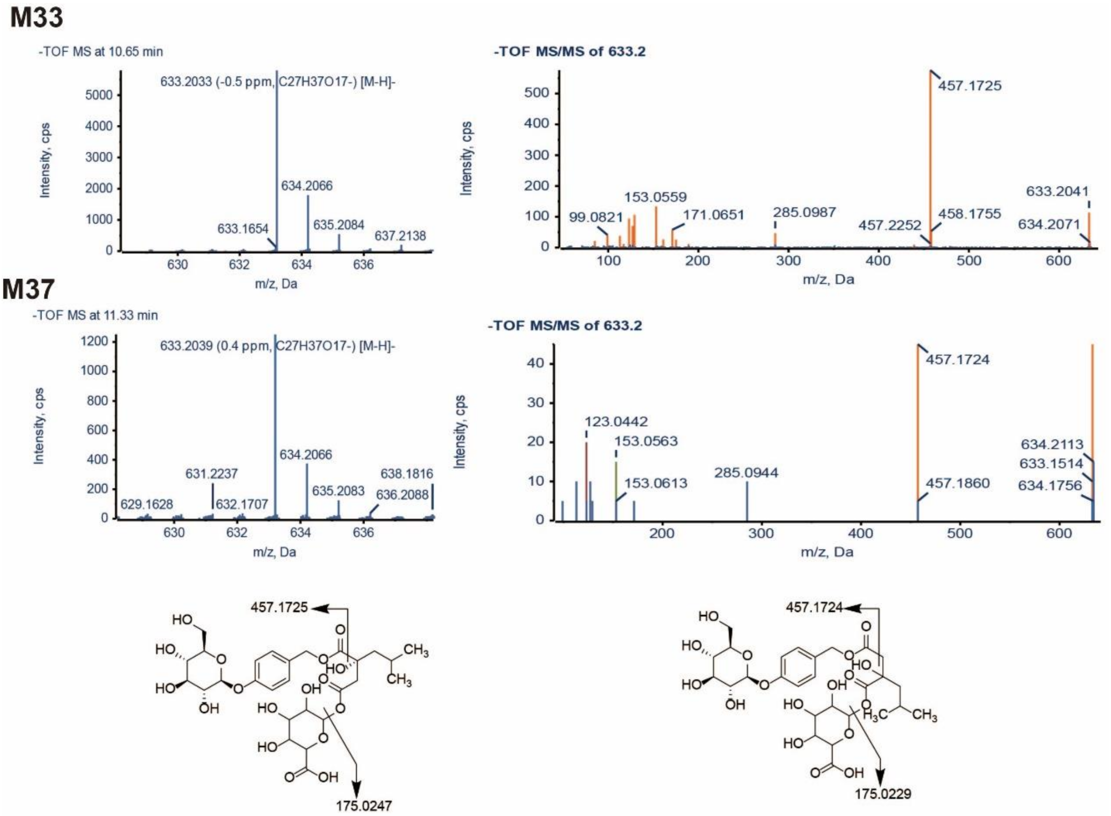

Figure S31. MS and MS/MS spectra of metabolites (M33, M37) in negative ESI mode and the proposed fragmentation pathway.

(32) metabolites (M34, M36) having the skeleton of glycosylated isobutyl-malic acid

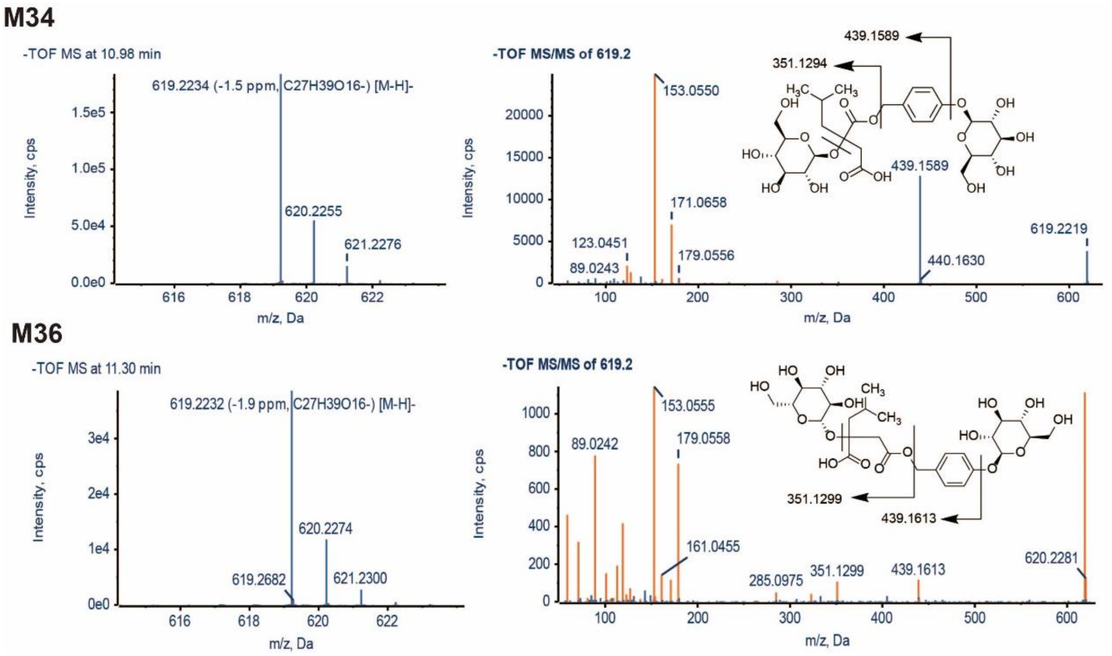

Figure S32. MS and MS/MS spectra of metabolites (M34, M36) in negative ESI mode and the proposed fragmentation pathway.

(33) metabolites (M35, M38, M41, M44, M46, M49) having the skeleton of trisaccharide

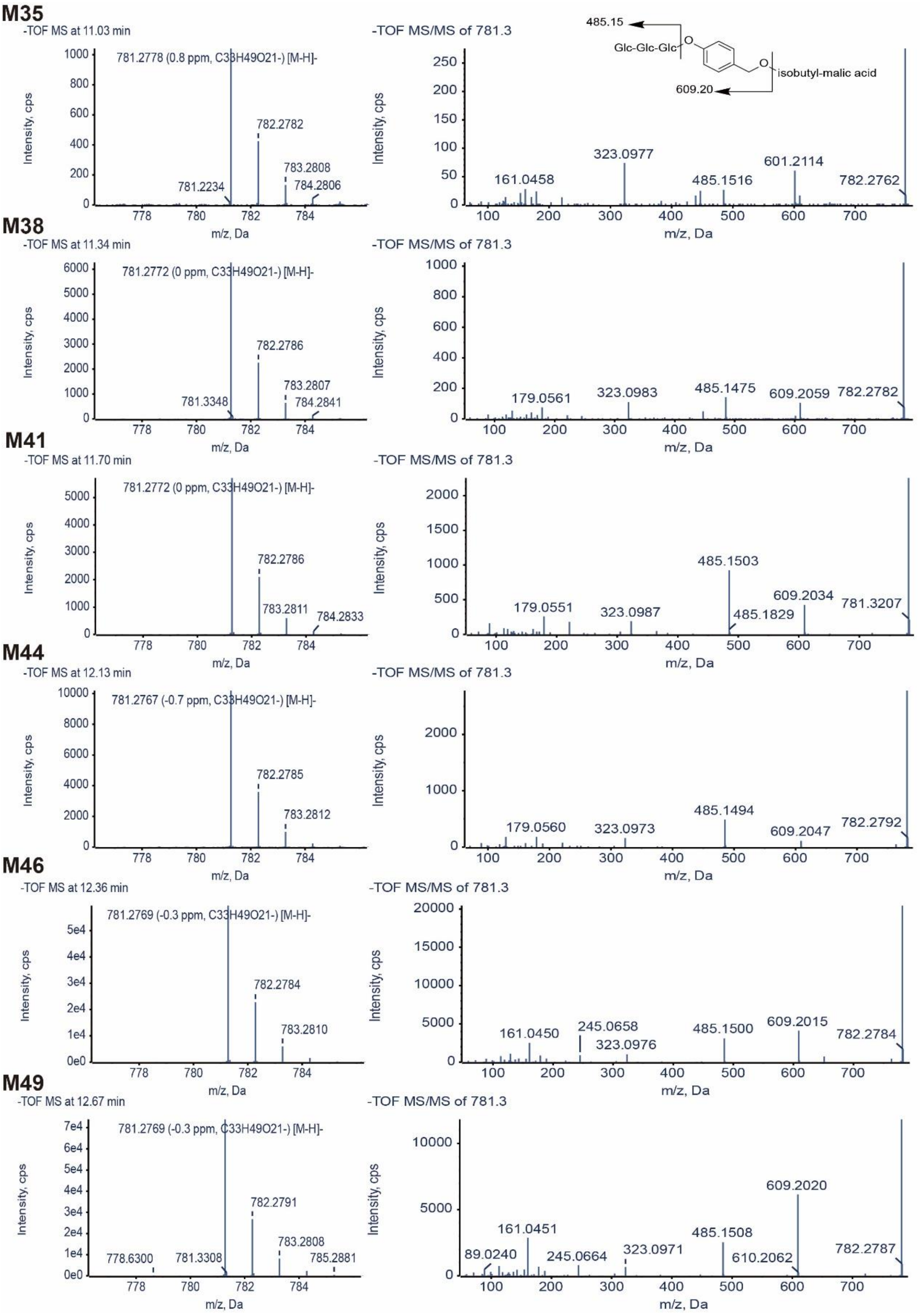

Figure S33. MS and MS/MS spectra of metabolites (M35, M38, M41, M44, M46, M49) in negative ESI mode and the proposed fragmentation pathway.

(34) metabolites (M39, M42, M43, M45, M47, M50, M54) having the fragment of disaccharide

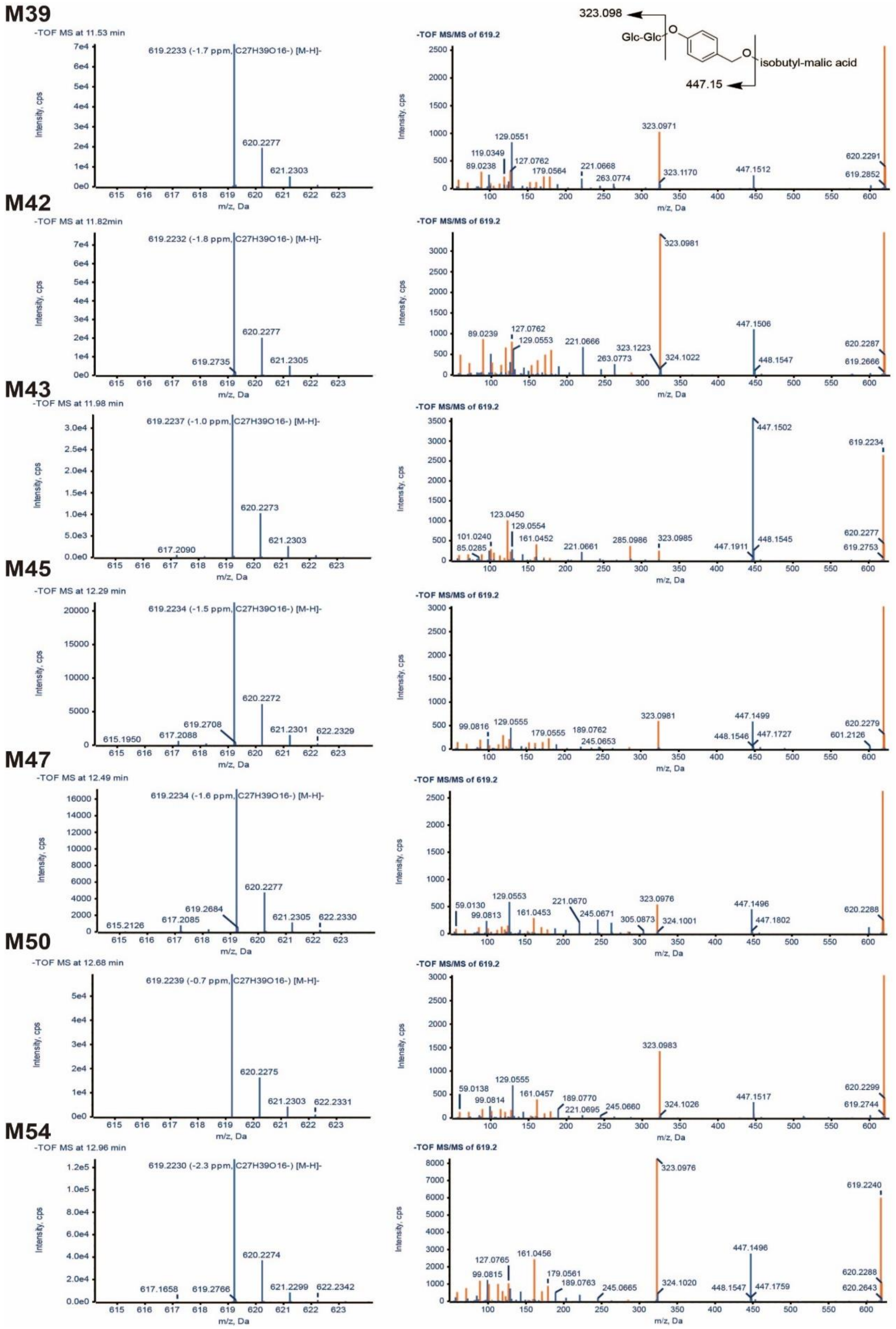

Figure S34. MS and MS/MS spectra of metabolites (M39, M42, M43, M45, M47, M50, M54) in negative ESI mode and the proposed fragmentation pathway.

(35) metabolites (M40, M48, M53) having the fragment of glucuronidated glucose

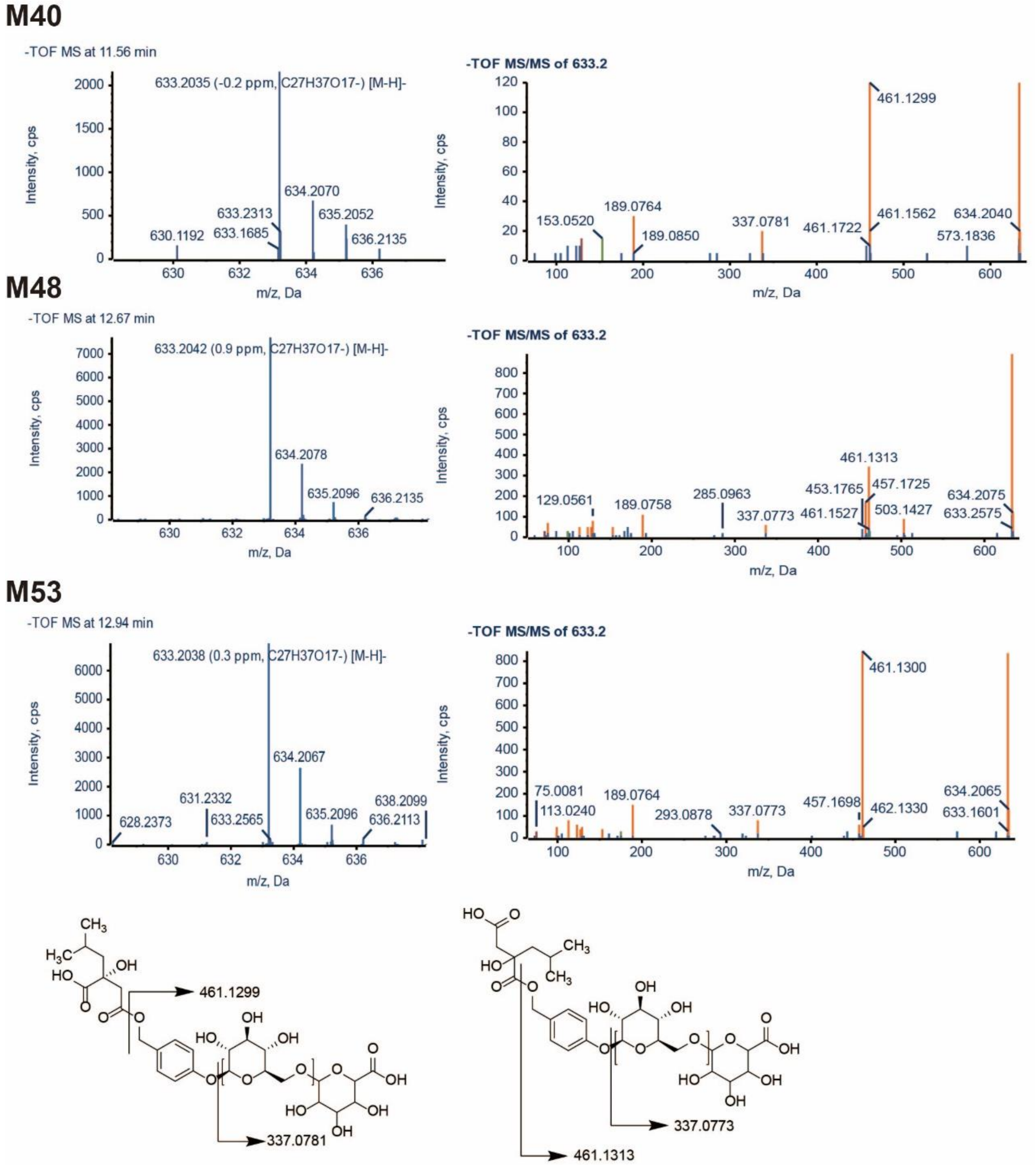

Figure S35. MS and MS/MS spectra of metabolites (M40, M48, M53) in negative ESI mode and the proposed fragmentation pathway.

(36) metabolites (M51, M56) having the fragment of gastrodin and isobutyl-malic acid

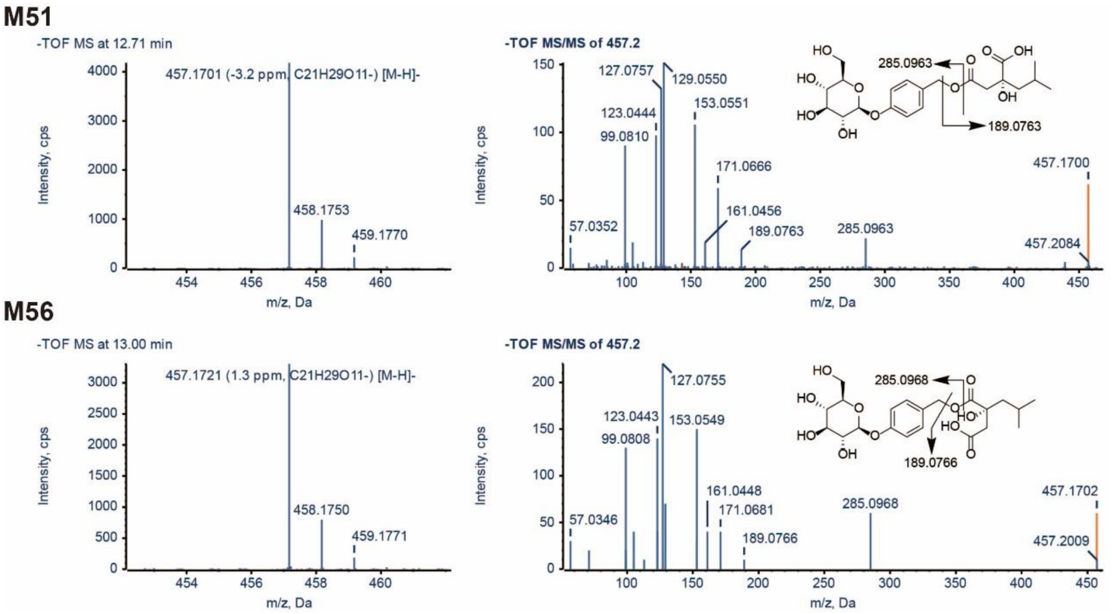

Figure S36. MS and MS/MS spectra of metabolites (M51, M56) in negative ESI mode and the proposed fragmentation pathway.

(37) metabolites (M52, M55) having the fragment of glucuronic acid

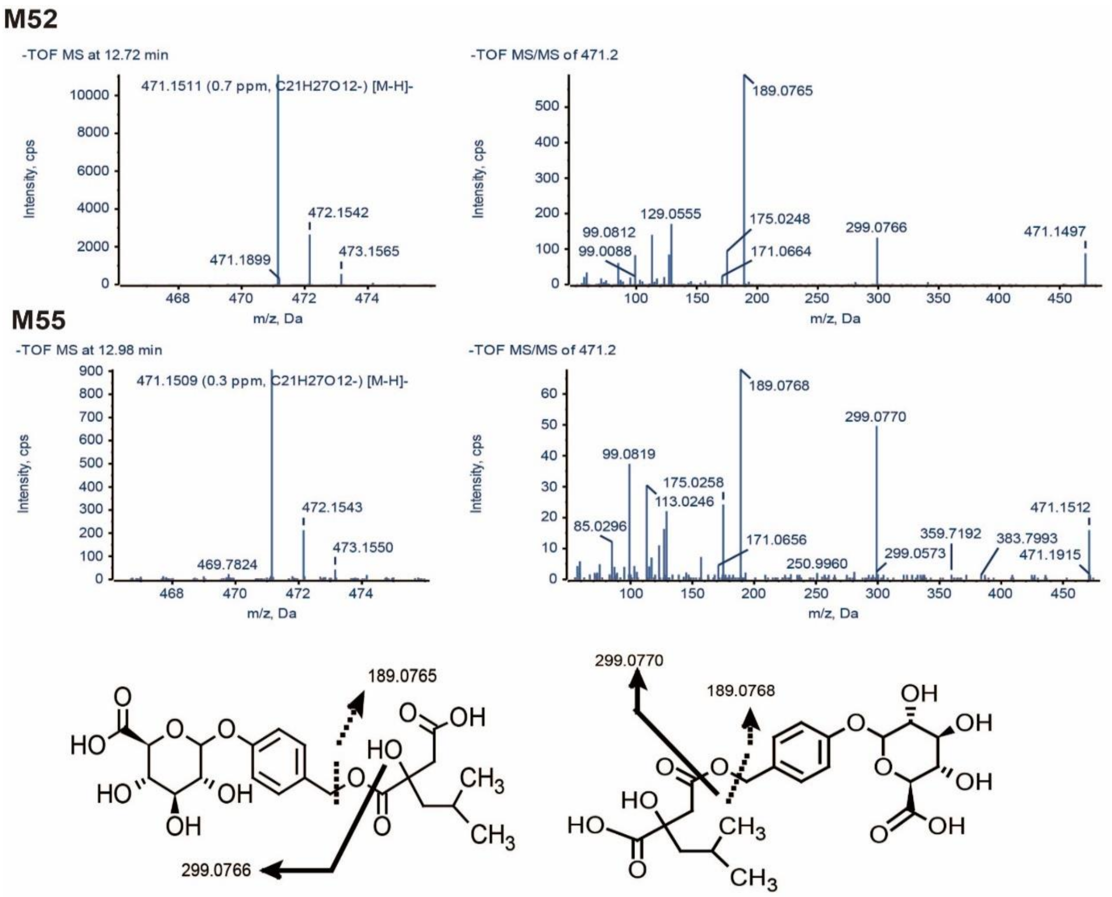

Figure S37. MS and MS/MS spectra of metabolites (M52, M55) in negative ESI mode and the proposed fragmentation pathway.

(38) metabolites (M57–M60) having the fragment of sulfated glucose

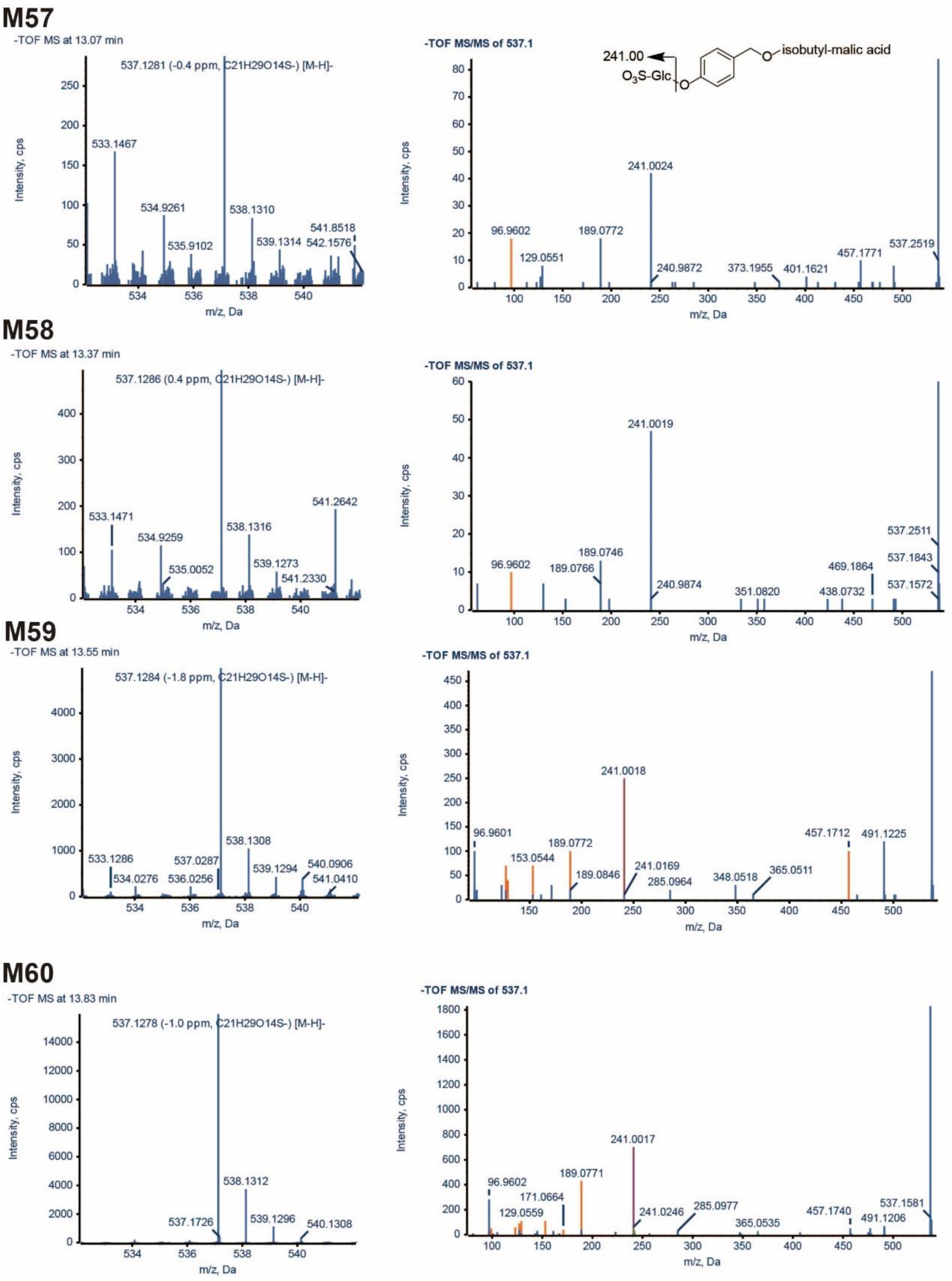

Figure S38. MS and MS/MS spectra of metabolites (M57 – M60) in negative ESI mode and the proposed fragmentation pathway.

(39) metabolites (M61, M66) having the fragment of glucuronidated p-hydroxybenzyl alcohol

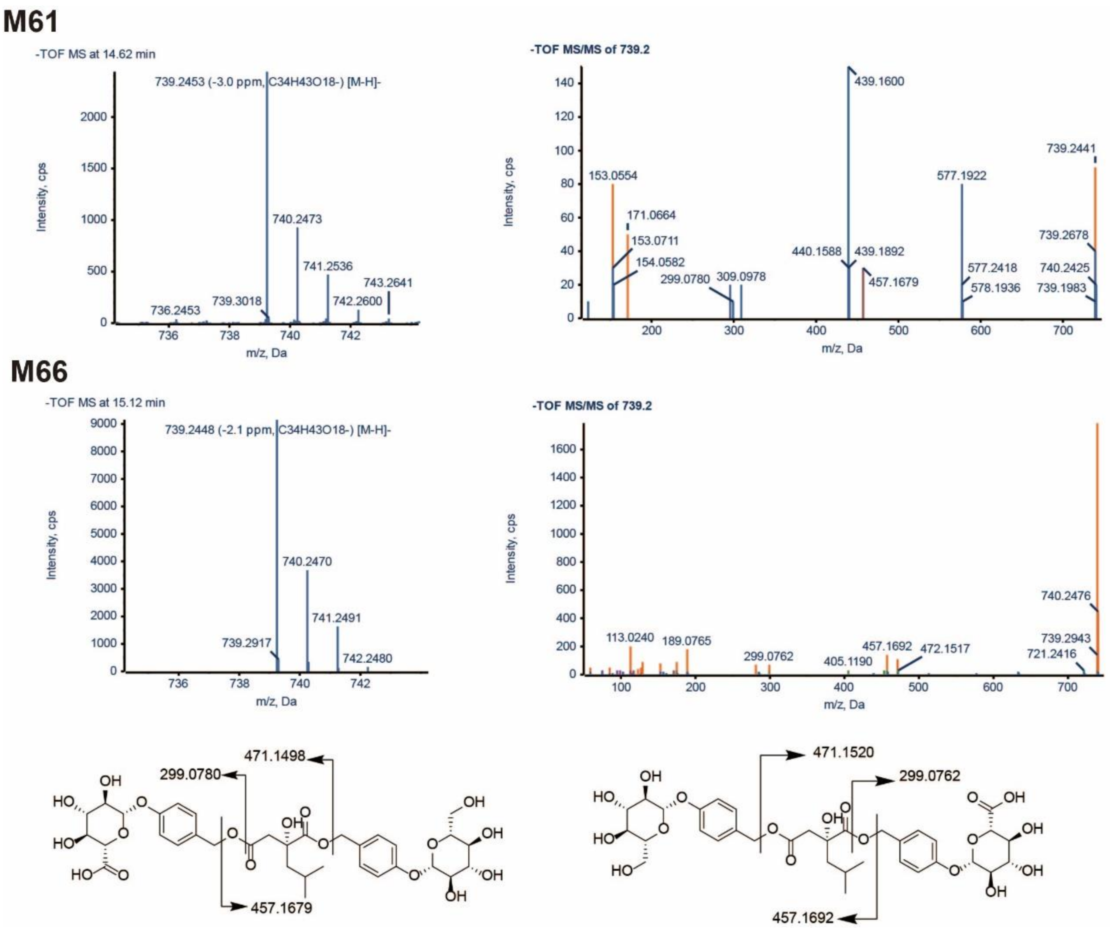

Figure S39. MS and MS/MS spectra of metabolites (M61, M66) in negative ESI mode and the proposed fragmentation pathway.

(40) metabolites (M62, M63) having the fragment of hydroxylated gymnoside I(II)

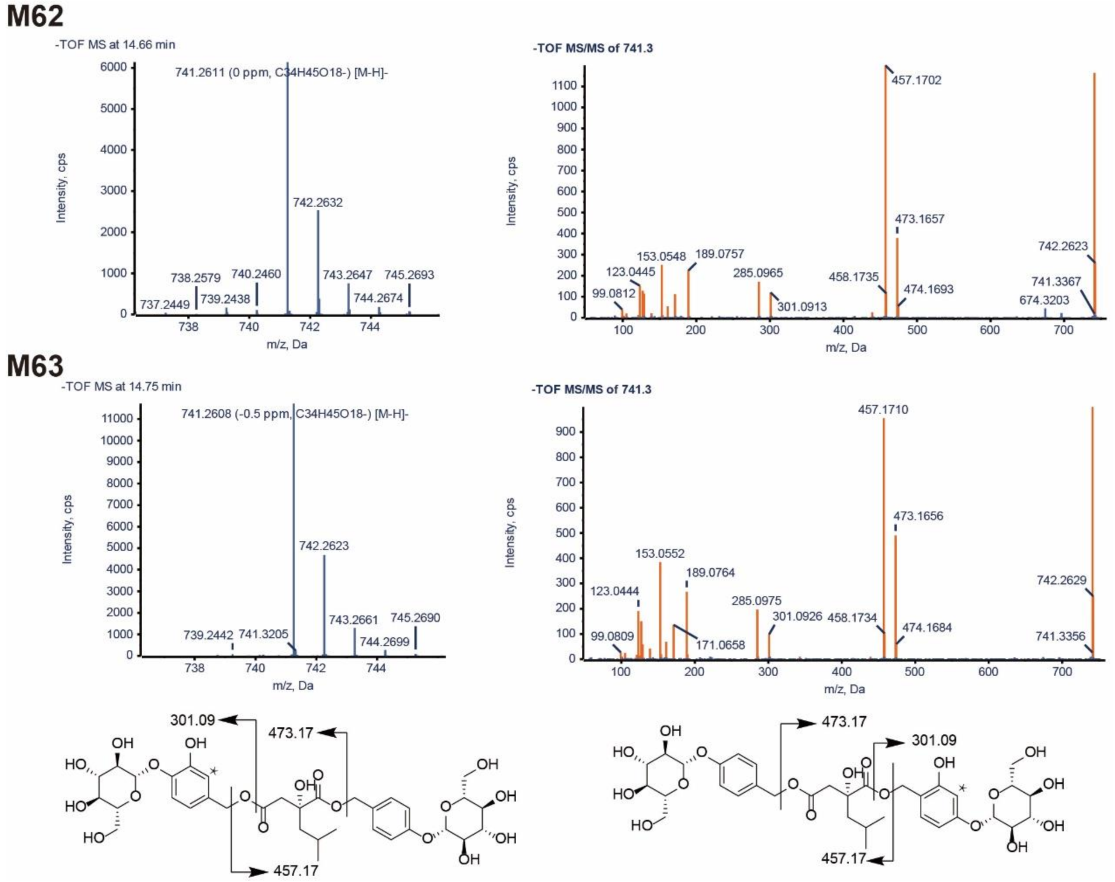

Figure S40. MS and MS/MS spectra of metabolites (M62, M63) in negative ESI mode and the proposed fragmentation pathway. * presents the alternative position of hydroxyl.

(41) metabolites (M64, M65) having the fragments of glycosylated gymnoside I(II) and disaccharide

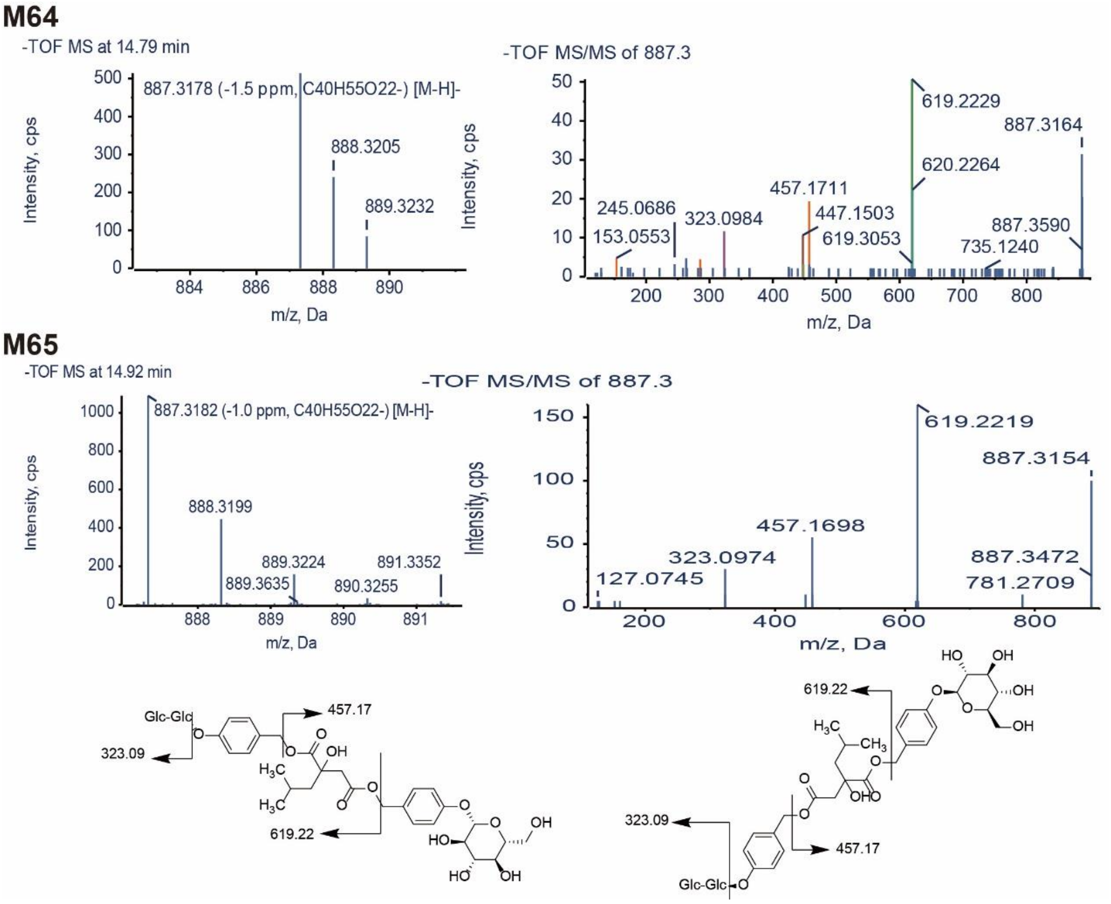

Figure S41. MS and MS/MS spectra of metabolites (M64, M65) in negative ESI mode and the proposed fragmentation pathway.

(42) metabolites (M67–M69) having the fragments of having the fragments of glycosylated gymnoside I(II) and disaccharide

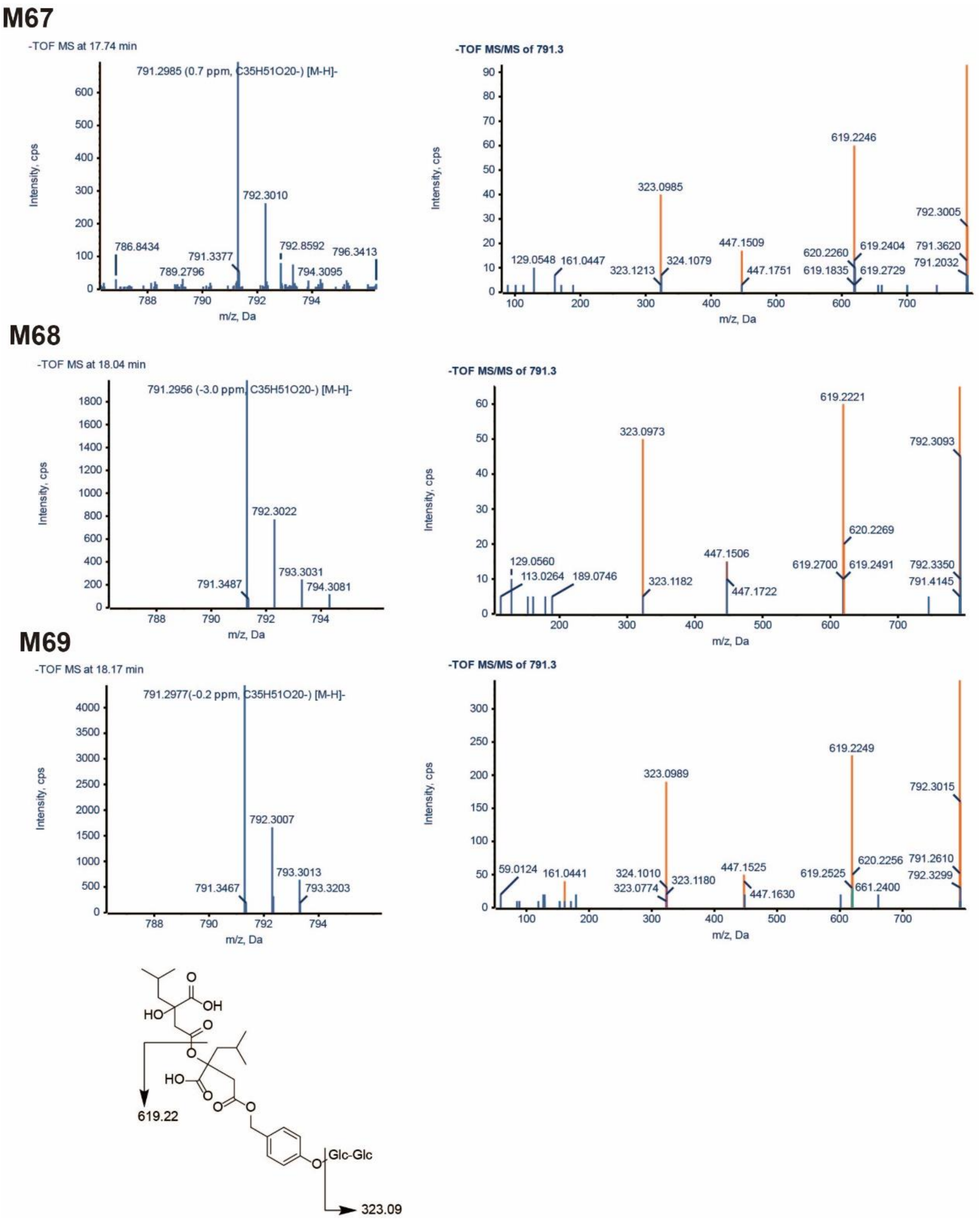

Figure S42. MS and MS/MS spectra of metabolites (M67 – M69) in negative ESI mode and the proposed fragmentation pathway.

(43) metabolites (M70, M71) having the fragments of having the fragments of gymnoside I(II) and gastrodin

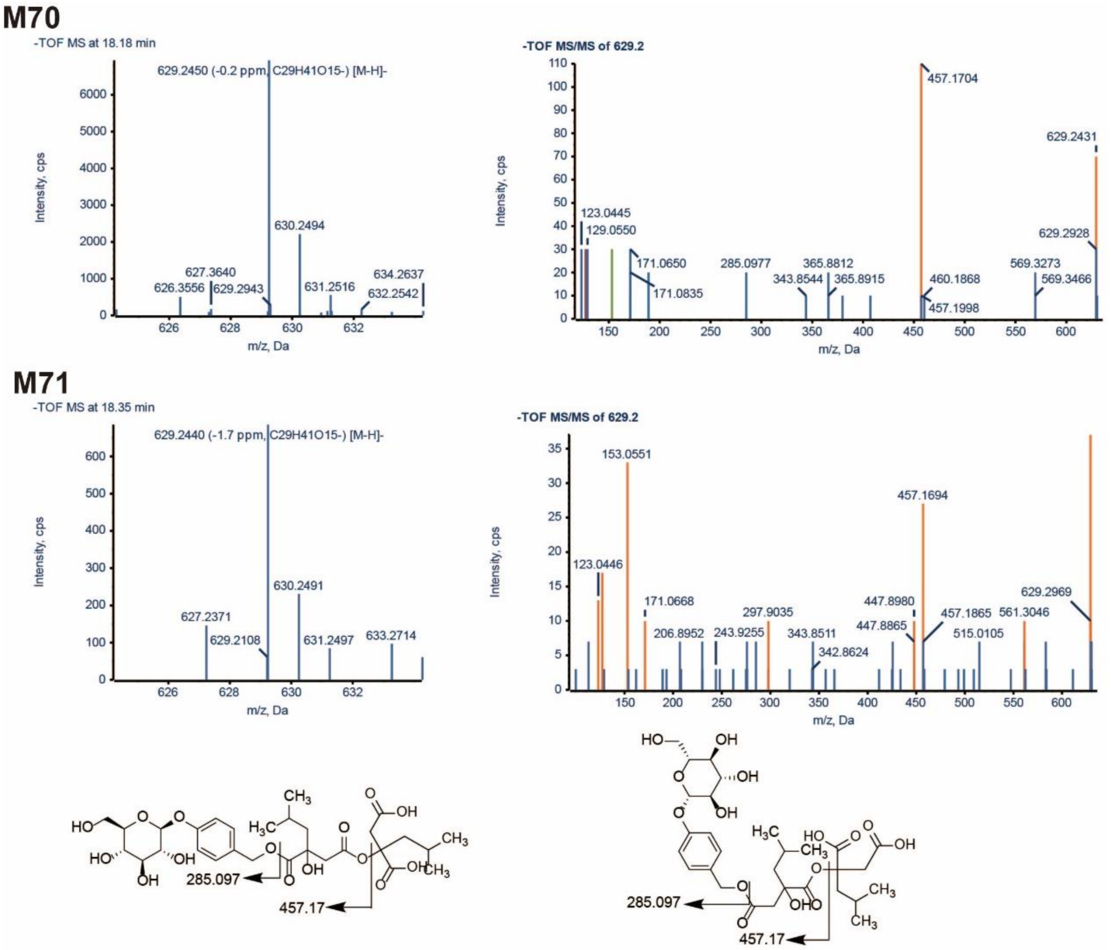

Figure S43. MS and MS/MS spectra of metabolites (M70, M71) in negative ESI mode and the proposed fragmentation pathway.
